# Clinical efficacy of tirzepatide for weight regain and suboptimal glycemic control following laparoscopic sleeve gastrectomy: a case series of three patients with obesity-associated type 2 diabetes

**DOI:** 10.3389/fendo.2026.1895420

**Published:** 2026-08-14

**Authors:** Midori Fujishiro, Ken Hagiwara, Arisa Nemoto, Hatsumi Kuroyanagi, Maiko Nosoko, Naoko Okamura, Urara Kon, Hiroko Uchida, Katsuhiko Ogawa, Hisamitsu Ishihara

**Affiliations:** 1 Division of Diabetes and Metabolic Diseases, Department of Internal Medicine, Nihon University School of Medicine, Tokyo, Japan; 2 Department of Internal Medicine, Nihon University Hospital, Tokyo, Japan; 3 Department of Gastrointestinal Surgery, Nihon University Hospital, Tokyo, Japan; 4 Division of General Medicine, Department of Internal Medicine, Nihon University School of Medicine, Tokyo, Japan; 5 Department of Nutrition Management, Nihon University Hospital, Tokyo, Japan

**Keywords:** glucagon-like peptide-1 receptor agonists, laparoscopic sleeve gastrectomy, tirzepatide, type 2 diabetes mellitus, weight regain after bariatric surgery

## Abstract

**Background:**

Laparoscopic sleeve gastrectomy is an effective metabolic surgery; however, some patients experience weight regain and worsening of type 2 diabetes mellitus (T2DM) over the long term. Tirzepatide (TZP), a dual glucose-dependent insulinotropic polypeptide (GIP) and glucagon-like peptide-1 (GLP-1) receptor agonist, has demonstrated potent weight loss and glucose-lowering effects. This study evaluated the clinical outcomes of TZP treatment in patients facing weight regain and insufficient glycemic control after LSG.

**Methods and results:**

We performed a retrospective clinical analysis of three male patients with obesity-associated T2DM who developed significant weight regain and suboptimal glycemic control several years after undergoing LSG. Despite repeated nutritional counseling and behavioral modification, their conditions remained refractory. Following the initiation of weekly TZP treatment, all three patients achieved substantial weight reduction and significant improvements in HbA1c levels. Specifically, Case 1 achieved a total weight loss (%TWL) of 14.0% from the pre-LSG baseline (13.2% weight loss specifically from TZP initiation) over 12 months. Case 2 achieved a %TWL of 13.2% from the pre-LSG baseline (8.5% weight loss from TZP initiation) over 6 months. Case 3 achieved a %TWL of 27.3% from the pre-LSG baseline (28.7% weight loss from TZP initiation) over 9 months. Notably, all patients reached HbA1c levels below 6.0% (range: 5.2%–5.6%), surpassing the glycemic control achieved in the immediate postoperative period. TZP was well-tolerated, with only mild constipation reported as an adverse event.

**Conclusion:**

TZP may serve as a promising therapeutic option for managing weight regain and insufficient glycemic control in patients after metabolic bariatric surgery, potentially delaying or eliminating the need for complex revisional surgery.

## 1 Introduction

In Japan, individuals with a body mass index (BMI) of 25.0 kg/m² or higher are diagnosed with obesity, whereas the WHO and the National Institutes of Health (NIH) in the USA define obesity as a BMI ≥30 kg/m² (1, 2). This lower threshold is used because obesity-related complications in Japanese subjects increase at a BMI ≥25 kg/m² (3). Furthermore, individuals with obesity-associated complications and/or visceral fat accumulation are diagnosed with “obesity disease,” indicating a medical necessity for weight reduction. Because obesity with 25 ≤ BMI < 35 and obesity with BMI ≥ 35 differ in both pathophysiology and required therapeutic approaches, those with a BMI ≥ 35 are categorized and managed separately as having “high-degree obesity.” If they present with obesity-related health disorders or visceral fat accumulation, they are diagnosed with “high-degree obesity disease” (4).

Type 2 diabetes (T2DM) is a major comorbidity related to obesity, particularly high-degree obesity. Controlling both conditions remains challenging through conventional medical treatments, including diet, exercise, behavioral modification, and medications. Metabolic bariatric surgery (MBS) is currently the most effective treatment for high-degree obesity disease, providing durable weight loss that leads to the control of obesity-related comorbidities—especially T2DM—while improving cardiovascular risk factors and reducing all-cause mortality (5, 6). However, weight loss insufficiency or weight regain occurs at a certain rate, whether immediately or following initial success (7–10). To date, a standard therapeutic strategy for weight regain after MBS has yet to be established. One option is surgical revision to treat weight regain and achieve T2DM remission; however, this approach is associated with a higher complication rate than the initial surgery (11–13).

Glucagon-like peptide-1 receptor agonists (GLP-1 RAs) are currently the most effective anti-obesity drugs and were initially developed as anti-diabetic medications. Among them, liraglutide and semaglutide are well-documented to achieve an average body weight loss of up to 15% along with cardiorenal benefits (14–16). They have also been reported as effective for weight regain in patients with or without T2DM after MBS (17–20). Tirzepatide (TZP), a dual glucose-dependent insulinotropic polypeptide (GIP) and GLP-1 RA, has been reported to be more effective for body weight loss than semaglutide—a selective GLP-1 RA—and is similarly beneficial in reducing major adverse cardiovascular events (21, 22). Recently, TZP was also documented as effective for weight regain after MBS (23); however, clinical evidence remains limited.

In this report, we describe three patients with obesity-associated T2DM who were effectively treated with TZP for weight regain and insufficient glycemic control following laparoscopic sleeve gastrectomy (LSG).

## 2 Materials and methods

### 2.1 Study design and participants

This single-center, retrospective observational study reviewed the clinical findings and longitudinal laboratory data of three male patients with obesity-associated T2DM who underwent primary LSG and subsequently received TZP therapy for post-operative weight regain and insufficient glycemic control. Post-operative weight regain was defined in accordance with the International Federation for the Surgery of Obesity and Metabolic Disorders (IFSO) consensus guidelines as a ≥10% increase in body weight from the post-operative nadir (10). Additionally, based on established evidence that even modest weight regain after metabolic surgery is strongly associated with the recurrence of T2DM and clinical relapse (24), for the purpose of this case series, we clinically defined a progressive weight increase of <10% accompanied by significant glycemic deterioration (glycosylated hemoglobin [HbA1c] relapse) as ‘metabolic weight regain’ that warranted immediate pharmacological intervention in our clinical practice. To maintain strict clinical and mathematical clarity regarding weight changes, total weight loss percentage (%TWL) was evaluated using two distinct metrics throughout the manuscript (1): %TWL from the pre-LSG baseline, defined as the percentage of weight loss relative to the pre-surgical baseline weight, and (2) %TWL from TZP initiation, defined as the percentage of weight loss achieved specifically during the course of TZP therapy relative to the weight at the time of drug initiation. All chronological and clinical data across the abstract, text, and tables were thoroughly cross-checked to ensure absolute internal consistency. The pre-LSG baseline clinical findings and laboratory data of the three cases are presented in **Tables 1** and **2**, respectively.

**Table 1:** Clinical findings at the pre-LSG baseline of the three cases.

Variable	Case 1	Case 2	Case 3
Sex	male	male	male
Age (years)	50	66	54
Body height (cm)	176	182.5	165.1
Body weight (kg)	108.4	143.4	119.6
BMI (kg/m2)	35	43.1	43.9
Alcohol	Never	Social	Never
Smoking	Former smoker	Former smoker	Former smoker
Type of diabetes	type 2	type 2	type 2
Duration of diabetes	12 years	6 years	9 years
Retinopathy	Preproliferative state	No retinopathy	Preproliferative state
Nephropathy	Microalbuminuria	Pre-nephropathy	Pre-nephropathy
Neuropathy	Polyneuropathy	Absent	Polyneuropathy
Other Complications	Hypertension	Hypertension	Hypertension
	Dyslipidemia	Dyslipidemia	Dyslipidemia
	Steatotic liver disease	Steatotic liver disease	Steatotic liver disease
	Obstructive sleep Apnea Syndrome	Obstructive sleep Apnea Syndrome	Obstructive sleep Apnea Syndrome
	Hyperuricemia	Cervical disc herniation	
Past history	Pulmonary vein isolation	Cholecystectomy	Percutaneous Coronary Intervention
	Cerebral infarction		
Medication for diabetes	1.8 mg daily of liraglutide	2,250 mg daily of metformin	2,250 mg daily of metformin
	1,500 mg daily of metformin		100 mg daily of canagliflozin
			0.9 mg daily of voglibose

BMI, body mass index.

**Table 2:** Laboratory data of the three cases at the pre-LSG baseline.

Variable	Reference range	Case 1	Case 2	Case 3
Peripheral blood				
White blood cells (/μL)	3300–8600	10,500	3,100	5,800
Neutrophil (%)	40.0–70.0	57.7	53.7	63.8
Red blood cells (× 104/μL)	386–492	516	470	524
Hemoglobin (g/dL)	11.6–14.8	15	15.5	15.7
Hematocrit (%)	35.1–44.4	45.1	45.4	46.4
Platelets (× 104/μL)	15.8–34.8	36.2	16.0	17.9
Blood biochemistry				
Total protein (g/dL)	6.6–8.1	8.1	6.9	7.3
Albumin (g/dL)	4.1–5.1	4.9	4.1	4.2
Total bilirubin (mg/dL)	0.4–1.5	0.55	2.22	0.88
AST (U/L)	13–30	23	33	36
ALT (U/L)	10-42	32	40	60
LDH (U/L)	124–222	131	155	168
ALP (U/L)	38–113	76	190	64
γ-GTP (U/L)	13-64	42	72	60
Cholinesterase (U/L)	240-486	343	322	291
Amylase (U/L)	44-132	83	108	50
BUN (mg/dL)	8–20	22.7	10.2	10.6
Creatinine (mg/dL)	0.46–0.79	1.36	0.86	0.82
Uric acid (mg/dL)	2.6–7.0	7.2	6.2	5.7
CRP (mg/dL)	<0.200	0.1	0.09	0.06
Sodium (mEq/L)	138–145	141	140	140
Potassium (mEq/L)	3.6–4.8	4.4	4.2	4.0
Chloride (mEq/L)	101–108	103	105	105
Diabetes and dyslipidemia markers				
Fasting Glucose (mg/dL)	73-109	114	133	115
Immunoreactive Insulin (μU/mL)	2.0-16.0	9.2	9.3	8.2
anti-GAD Antibody (U/mL)	<5.0	<5.0	<5.0	<5.0
HbA1c (%)	4.6–6.2	9.2	6.6	6.6
Total cholesterol (mg/dL)	142–219	164	150	136
HDL cholesterol (mg/dL)	40-90	33	39	42
Triglycerides (mg/dL)	30-149	278	104	129
Urinary test				
Urinary pH	5.0–7.0	5.5	7.0	5.0
Urinary protein	–	2+	–	–
Urinary sugar	–	–	–	–
Urinary ketone body	–	–	–	–
Urinary blood	–	–	–	–
Urine albumin-creatinine ratio (mg/gCre)	1.9-13.3	156.6	4.6	13.0

AST, aspartate aminotransferase; ALT, alanine aminotransferase; LDH, lactate dehydrogenase; ALP, alkaline phosphatase; γ-GTP, γ-glutamyl transpeptidase; BUN, blood urea nitrogen; CRP, C-reactive protein; GAD, glutamic acid decarboxylase; HbA1c, glycosylated hemoglobin; HDL, high-density lipoprotein.

### 2.2 Multidisciplinary guidance and tirzepatide titration protocol

All three patients received structured preoperative and ongoing postoperative guidance, including detailed dietary counseling from registered dietitians at the Department of Nutrition Management, exercise instruction from physical therapists, and behavioral modification therapy from their attending physicians. Despite these continuous multidisciplinary efforts, their weight and diabetes management gradually deteriorated after an initial period of success.

To manage weight regain, subcutaneous TZP therapy was initiated. For all cases, TZP was initiated at a dose of 2.5 mg weekly and titrated up by 2.5 mg every 4 weeks to a maximum maintenance dose of 15 mg weekly.

### 2.3 Ethical considerations

The study was conducted according to the guidelines of the Declaration of Helsinki and approved by the Institutional Ethics Committee of Nihon University Hospital (approval number 20220101). Written informed consent was obtained from all three subjects described in this report for the publication of their data and associated clinical outcomes.

## 3 Results

### 3.1 Case presentation 1

**Figure 1:**
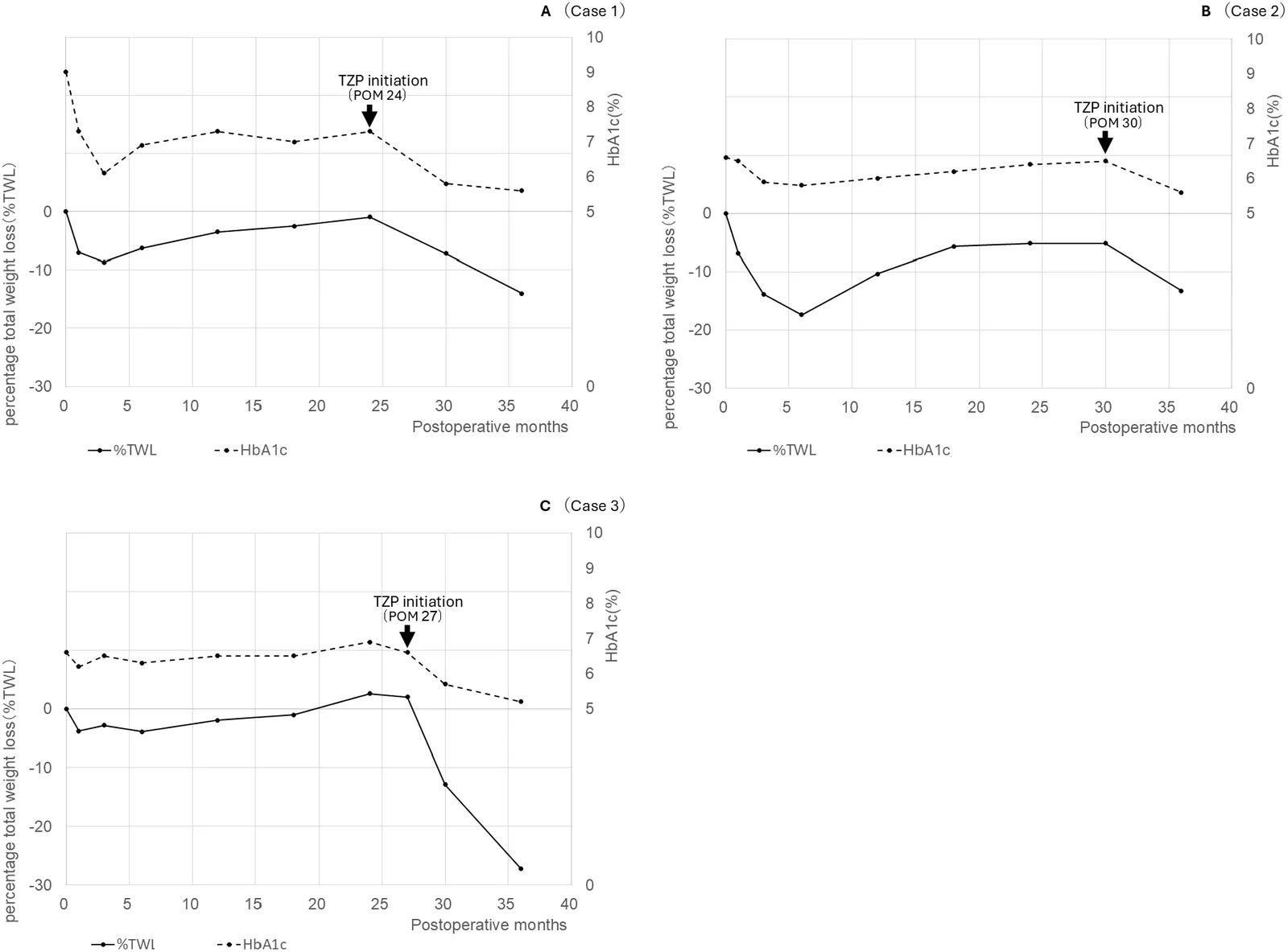
Longitudinal clinical courses of the three cases following LSG. The horizontal axis shows the time (months) since the date of laparoscopic sleeve gastrectomy (LSG). The left vertical axis represents the percentage total weight loss (%TWL, solid lines), where negative values indicate weight reduction relative to the pre-LSG baseline, and the right vertical axis represents glycosylated hemoglobin levels (HbA1c, %, dashed lines). **(A)** Case 1, **(B)** Case 2, and **(C)** Case 3. Significant post-operative weight regain and glycemic deterioration prompted the initiation of weekly tirzepatide (TZP) therapy.

A 50-year-old man (height, 176.0 cm) with a past medical history of T2DM, obesity, hypertension (HTN), dyslipidemia (DL), obstructive sleep apnea syndrome (OSAS), steatotic liver disease (SLD), history of cerebral infarction, and atrial fibrillation status post-pulmonary vein isolation. At the pre-LSG baseline, he was treated with liraglutide (1.8 mg/day) and metformin (1,500 mg/day), with a body weight of 108.4 kg, BMI of 34.9 kg/m², and HbA1c of 9.0%. At one month postoperatively (POM1), while on metformin (2,000 mg/day) monotherapy, his weight was 100.8 kg (a %TWL of 7.0% from the pre-LSG baseline), BMI was 32.4 kg/m², and HbA1c was 7.3%. Although his HbA1c briefly improved to 6.9% at POM14, it subsequently rose back to 7.3% at POM24, coinciding with weight regain (107.4 kg, representing an 8.5% increase from his post-operative nadir weight of 99.0 kg achieved at POM3), and TZP was initiated. At the initiation of TZP, his HbA1c had deteriorated to 7.3%, compared to his post-operative best of 6.1% at POM3. By POM36, following treatment with TZP, he achieved a body weight of 93.2 kg, representing a %TWL of 14.0% from the pre-LSG baseline and a %TWL of 13.2% specifically achieved during the 12-month TZP therapy (weight decreased from 107.4 kg at TZP initiation to 93.2 kg), with a BMI of 30.0 kg/m², and HbA1c of 5.6% (**Figure 1**). Notably, one month after reaching the maintenance dose of TZP (15 mg), metformin (750 mg/day) was discontinued. Under our multidisciplinary guidance, the patient reported a subjective decrease in appetite, improved portion control, and enhanced adherence to physical activity (walking during commute). Longitudinal body composition analysis via BIA (MC-780MA-N, Tanita Corp., Tokyo, Japan) revealed a reduction in body fat percentage from 39.4% (TZP initiation) to 35.0% (post-TZP), while his muscle mass-to-body weight ratio improved from 57.4% to 61.7% (absolute muscle mass changed from 61.7 kg to 57.5 kg), demonstrating that skeletal muscle mass appeared largely preserved relative to total weight loss. His diabetic nephropathy also improved, with the urine albumin-to-creatinine ratio (UACR) decreasing from 156.6 mg/gCre (pre-LSG) to 48.3 mg/gCre at POM36.

### 3.2 Case presentation 2

A 66-year-old man (height, 182.5 cm) with a history of T2DM, obesity, HTN, DL, OSAS, SLD, cervical disc herniation, and status post-cholecystectomy. At the pre-LSG baseline, he received metformin (2,250 mg/day), with a body weight of 143.4 kg, BMI of 43.1 kg/m², and HbA1c of 6.6%. At POM1, without antidiabetic medication, his weight was 133.5 kg (a %TWL of 6.9% from the pre-LSG baseline), BMI was 40.1 kg/m², and HbA1c was 6.5%. At POM30, his weight increased to 136.0 kg (representing a 14.8% increase from his post-operative nadir weight of 118.5 kg achieved at POM6), BMI to 40.8 kg/m², and HbA1c remained at 6.5% (compared to his post-operative best of 5.8% at POM6), at which point TZP was initiated. By POM36, following treatment with TZP, his weight stabilized at 124.4 kg, representing a %TWL of 13.2% from the pre-LSG baseline and a %TWL of 8.5% specifically achieved during the 6-month TZP therapy (weight decreased from 136.0 kg at TZP initiation to 124.4 kg), with a BMI of 37.4 kg/m² and HbA1c of 5.6%, as shown in the clinical course in **Figure 1**. His baseline antidiabetic medications were maintained without further escalations. He reported successful portion control under dietary guidance, though physical exercise intensification was restricted due to his cervical disc herniation. BIA analysis demonstrated a reduction in body fat percentage from 46.0% to 42.1%, with an improvement in his muscle mass-to-body weight ratio from 51.2% to 54.9% (absolute muscle mass changed from 69.6 kg to 68.3 kg).

### 3.3 Case presentation 3

A 54-year-old man (height, 165.1 cm) with a history of T2DM, obesity, HTN, DL, OSAS, SLD and old myocardial infarction. Pre-LSG medications included canagliflozin (100 mg/day), metformin (2,250 mg/day), and voglibose (0.9 mg/day); his weight was 119.6 kg, BMI was 43.9 kg/m², and HbA1c was 6.6%. At POM1, on metformin (1,500 mg/day) monotherapy, his weight was 115.0 kg (a %TWL of 3.8% from the pre-LSG baseline), BMI was 42.2 kg/m², and HbA1c was 6.2%. By POM24, his clinical status had deteriorated to a peak body weight of 122.7 kg (a 6.8% increase from his post-operative nadir weight of 114.9 kg achieved at POM6) and a peak HbA1c of 6.9% (compared to his post-operative best of 6.2% at POM1). At this juncture, due to the clear clinical relapse, pharmacological intervention with weekly TZP was proposed to the patient. Following clinical preparation and treatment planning, TZP was formally initiated at POM27, at which point his weight was 122.0 kg (a 6.2% increase from nadir) and HbA1c was 6.6%. At POM36, following treatment with TZP, he achieved a significant weight reduction to 87.0 kg, representing a %TWL of 27.3% from the pre-LSG baseline and a %TWL of 28.7% specifically achieved during the 9-month TZP therapy (weight decreased from 122.0 kg at TZP initiation to 87.0 kg), with a BMI of 31.9 kg/m², and HbA1c of 5.2% (**Figure 1**). His baseline antidiabetic medications were maintained without adjustments. Under lifestyle guidance, the patient reported a significant reduction in cravings and snack frequency, achieving high adherence to physical exercise by walking approximately 3 hours daily. BIA analysis revealed a marked reduction in body fat percentage from 47.7% to 30.1%, accompanied by a substantial increase in his muscle mass-to-body weight ratio from 49.6% to 66.3% (absolute muscle mass changed from 60.5 kg to 57.7 kg).

### 3.4 Clinical course following LSG and safety outcomes

Following MBS, the patients initially achieved successful weight reduction and improved glycemic control, but gradually deteriorated despite continued nutritional counseling and behavioral guidance. The primary factors contributing to this weight regain included an increase in food intake and a lack of regular physical activity driven by demanding work schedules. Upon initiation of TZP, all patients reported enhanced satiety and a reduction in the volume of food consumed per meal, which facilitated successful weight loss and improved glycemic stabilization following their post-MBS weight regain. Regarding safety, TZP was well-tolerated; except for mild constipation, no serious adverse events were detected among the three subjects.

## 4 Discussion

MBS, particularly LSG, remains one of the most effective interventions for severe obesity and its related comorbidities. However, a subset of patients inevitably experiences weight regain and subsequent deterioration of glycemic control during long-term follow-up (25). While surgical revision is an option, it carries a higher risk of complications. Our case series demonstrates that TZP can be a potent pharmacological adjunct to manage these challenges effectively.

### 4.1 Synergistic effects of GIP and GLP-1 receptor agonism

The notable efficacy observed in our patients—particularly the significant weight reduction in Case 3 (29.3% over 9 months)—likely stems from the dual agonism of GIP and GLP-1 receptors. While GLP-1 RAs primarily reduce energy intake by delaying gastric emptying and enhancing satiety, GIP acts on the hypothalamus to further regulate appetite and may improve systemic lipid metabolism and insulin sensitivity in adipose tissue (26). In the post-LSG setting, where endogenous GLP-1 and peptide YY, levels are already altered (27), the addition of TZP may provide a complementary metabolic stimulus that overcomes the plateau or “escape” from initial surgical benefits. Furthermore, the reduction in UACR observed in Case 1 suggests that TZP may provide additional renoprotective benefits beyond weight loss, consistent with the findings from the SURPASS clinical trials (28, 29). Consistent with these findings, the initiation of TZP in our series was associated with noticeable longitudinal improvements in systolic/diastolic blood pressure and lipid profiles (as detailed in **Table 3**), further supporting the comprehensive cardiometabolic and cardiorenal benefits of dual GIP/GLP-1 receptor agonism in this high-risk population.

**Table 3:** Changes in weight metrics, blood pressure, lipid profiles, and body composition parameters before and after TZP therapy.

Parameter	Case 1	Case 2	Case 3
Weight Metric			
%TWL from pre-LSG baseline (%)			
At TZP initiation	0.9	5.2	-2.0
At POM 36 (Post-TZP)	14.0	13.2	27.3
Blood pressure (mmHg)			
At TZP initiation	137/97	149/98	169/104
At POM 36 (Post-TZP)	137/87	137/84	126/80
Lipid profile			
Total cholesterol (mg/dL)			
At TZP initiation	211	157	139
At POM 36 (Post-TZP)	171	131	134
HDL cholesterol (mg/dL)			
At TZP initiation	40	49	53
At POM 36 (Post-TZP)	43	46	62
LDL cholesterol (mg/dL)			
At TZP initiation	141	89	60
At POM 36 (Post-TZP)	108	69	53
Triglycerides (mg/dL)			
At TZP initiation	199	93	97
At POM 36 (Post-TZP)	103	61	59
Body composition (BIA)			
Body Fat Percentage (%)			
At TZP initiation	39.4	46.0	47.7
At POM 36 (Post-TZP)	35.0	42.1	30.1
Skeletal muscle Mass (kg)			
At TZP initiation	61.7	69.6	60.5
At POM 36 (Post-TZP)	57.5	68.3	57.7
Muscle Mass-to-Body Weight ratio (%)			
At TZP initiation	57.4	51.2	49.6
At POM 36 (Post-TZP)	61.7	54.9	66.3

### 4.2 Clinical course and patient-specific outcomes

All three patients reported improved satiety and a better ability to adhere to dietary modifications following TZP initiation. Case 1 showed a steady14.0% reduction in %TWL from pre-LSG baseline at POM 36, stabilizing his HbA1c at 5.6%. Case 2 achieved a 13.2% reduction in %TWL from pre-LSG baseline at POM 36, with his HbA1c reaching 5.6%. Case 3 demonstrated the most substantial response, achieving a 27.3% reduction in %TWL from pre-LSG baseline at POM 36 and an HbA1c of 5.2%. This is particularly noteworthy given his history of percutaneous coronary intervention and multiple oral antidiabetic drugs in the pre-LSG period. Notably, all three cases reached their lowest HbA1c levels (range: 5.2%–5.6%) only after starting TZP, surpassing the glycemic control achieved in the immediate post-operative period.

Recent clinical trials and cohort studies have increasingly supported the use of GLP-1-based therapies for managing post-bariatric weight recurrence. For instance, the landmark Bari-Optimise randomized clinical trial demonstrated that liraglutide 3.0 mg significantly improved weight loss and metabolic parameters compared to placebo in patients with insufficient weight loss or regain after metabolic surgery (19). More recently, real-world retrospective studies have shown that newer-generation agents, such as semaglutide and TZP, yield even more pronounced weight reduction in post-bariatric settings, with TZP demonstrating superior efficacy in both weight loss and glycemic control compared to selective GLP-1 RAs (20, 23). However, the vast majority of these studies were conducted in Western cohorts characterized by extreme, morbid obesity (typically with a mean BMI >40 kg/m², categorized as Class III obesity). In contrast, our case series provides valuable real-world evidence of tirzepatide’s efficacy in East Asian patients with obesity-associated T2DM, covering a clinically important and diverse BMI spectrum.

While Case 1 represented a patient with international Class II obesity who was positioned just below the threshold for “high-degree obesity” under Japanese guidelines (BMI ≥35 kg/m²) and maintained a stable Class II BMI range throughout (pre-LSG BMI 34.9 kg/m², TZP initiation BMI 34.6 kg/m²), Cases 2 and 3 represented patients with severe, international Class III morbid obesity starting from their initial consultation (pre-LSG BMI 43.1 kg/m² and 43.9 kg/m², respectively). Despite undergoing LSG, these patients experienced post-operative weight regain and metabolic relapse, with their BMI remaining within the Class III range at the time of TZP initiation (TZP initiation BMI 40.8 kg/m² and 44.8 kg/m², respectively). Given the distinct pathophysiology of East Asian patients, who exhibit limited pancreatic beta-cell capacity and develop severe metabolic complications at a lower BMI threshold, our findings highlight that dual GIP/GLP-1 receptor agonism can effectively overcome post-surgical metabolic escape and reverse weight regain and metabolic relapse across a wide clinical spectrum of obesity.

A major clinical concern during intensive weight loss with GIP/GLP-1 receptor agonists is the concomitant loss of lean muscle mass, which can increase the risk of sarcopenia. In our case series, routine BIA monitoring using the MC-780MA-N demonstrated that despite significant total weight loss, all three patients showed that absolute muscle mass appeared largely preserved with only minimal declines, while significantly improving their muscle mass-to-body weight ratio. This relative stability of muscle mass may be associated with the potent metabolic effects of tirzepatide combined with the patients’ strong adherence to lifestyle modifications, such as the intensive daily walking protocol in Case 3 and the commute-based physical activity in Case 1. These results suggest that structured multidisciplinary counseling combined with dual incretin therapy may be associated with favorable changes in body composition and weight loss in this clinical setting, though these preliminary findings warrant further investigation in larger prospective studies.

### 4.3 Tolerability and safety in post-LSG patients

A critical concern with GLP-1-based therapies after gastric surgery is gastrointestinal tolerability, as LSG itself significantly alters gastric anatomy and motility. In our series, TZP was well-tolerated; the only reported side effect was mild constipation, with no serious adverse events leading to treatment discontinuation. This suggests that the gradual dose titration of TZP allows the modified gastrointestinal tract to adapt effectively.

### 4.4 Limitations

The timing of TZP initiation and the active treatment duration differed among the three cases (12 months for Case 1, 6 months for Case 2, and 9 months for Case 3 by POM 36). This variability is an inherent limitation of our retrospective, real-world study design, where the initiation of pharmacotherapy was determined based on individual clinical presentation, glycemic relapse timing, and patient preference rather than a standardized protocol. Nevertheless, all three patients were longitudinally followed up to a consistent post-operative endpoint of POM 36. Future prospective trials with standardized treatment protocols and uniform follow-up periods are required to establish more robust clinical guidelines.

## 5 Conclusion

In conclusion, TZP represents a promising potential pharmacological adjunct for managing weight regain and insufficient glycemic control in patients after MBS. While these preliminary observations are encouraging, further prospective, comparative studies with larger cohorts are warranted to evaluate the long-term efficacy and safety of this approach in managing post-bariatric metabolic relapse.

## Data Availability

The original contributions presented in the study are included in the article. Further inquiries can be directed to the corresponding author.
